# The Role of Astrocytes in the Generation, Migration, and Integration of New Neurons in the Adult Olfactory Bulb

**DOI:** 10.3389/fnins.2016.00149

**Published:** 2016-04-05

**Authors:** Archana Gengatharan, Rodrigo R. Bammann, Armen Saghatelyan

**Affiliations:** ^1^Cellular Neurobiology Unit, Centre de Recherche de l'Institut Universitaire en Santé Mentale de QuébecQuebec, QC, Canada; ^2^Department of Psychiatry and Neuroscience, Université LavalQuebec, QC, Canada

**Keywords:** olfactory bulb, stem cells, astrocytes, interneurons, rostral migratory stream, blood vessels

## Abstract

In mammals, new neurons in the adult olfactory bulb originate from a pool of neural stem cells in the subventricular zone of the lateral ventricles. Adult-born cells play an important role in odor information processing by adjusting the neuronal network to changing environmental conditions. Olfactory bulb neurogenesis is supported by several non-neuronal cells. In this review, we focus on the role of astroglial cells in the generation, migration, integration, and survival of new neurons in the adult forebrain. In the subventricular zone, neural stem cells with astrocytic properties display regional and temporal specificity when generating different neuronal subtypes. Non-neurogenic astrocytes contribute to the establishment and maintenance of the neurogenic niche. Neuroblast chains migrate through the rostral migratory stream ensheathed by astrocytic processes. Astrocytes play an important regulatory role in neuroblast migration and also assist in the development of a vasculature scaffold in the migratory stream that is essential for neuroblast migration in the postnatal brain. In the olfactory bulb, astrocytes help to modulate the network through a complex release of cytokines, regulate blood flow, and provide metabolic support, which may promote the integration and survival of new neurons. Astrocytes thus play a pivotal role in various processes of adult olfactory bulb neurogenesis, and it is likely that many other functions of these glial cells will emerge in the near future.

## Introduction

The olfactory bulb (OB), which plays a central role in odor information processing, has a multi-layered cellular architecture. Mitral and tufted cells are the principal neurons of the OB (Shepherd et al., 2004) and transmit information received from olfactory sensory neurons to the piriform cortex, as well as the entorhinal cortex and the amygdala (Davis, 2004; Shepherd et al., 2004). Information processing in the OB is modulated by two other groups of interneurons, that is, periglomerular cells (PG) and granule cells (GC), which form dendro-dendritic synapses with the principal neurons (Urban, 2002; Fukunaga et al., 2014). Interestingly, a substantial number of PGs and GCs are constantly renewed during adulthood. This unusual form of plasticity, which was first brought to light some fifty years ago and was confirmed by subsequent research (Altman and Das, 1965; Altman, 1969; Luskin, 1993; Lois and Alvarez-Buylla, 1994; Doetsch et al., 1999), allows the OB circuitry to be fine-tuned in response to olfactory behaviors (Imayoshi et al., 2008; Breton-Provencher et al., 2009; Sultan et al., 2011; Alonso et al., 2012; Arruda-Carvalho et al., 2014). Many neurons arrive daily at the OB; therefore, it is necessary to tightly control the migration and neurochemical diversity of these cells, and to optimize the structure and function of the OB network by selectively controlling newborn cells survival and synaptic pruning. Astrocytes play a pivotal role in all these processes. In this review, we summarize the current knowledge of the role of astrocytes in adult OB neurogenesis and discuss possible future directions in this exciting field.

## Neurogenic and non-neurogenic astrocytes in the adult SVZ

### Neural stem cells

Adult neural stem cells (NSC) residing in the subventricular zone (SVZ), which are also called B1 cells, are derived from radial glial cells and have several astrocytic features. They express the glial fibrillary acidic (GFAP), glutamate aspartate transporter, and brain lipid-binding proteins (Codega et al., 2014; Fuentealba et al., 2015; Llorens-Bobadilla et al., 2015). They also present ultra structural properties of astrocytes, including a light cytoplasm, thick bundles of intermediate filaments, gap junctions, glycogen granules, and dense bodies (Jackson and Alvarez-Buylla, 2008). NSC can exist in either the quiescent or the active state. Once activated, GFAP-positive B1 cells express epidermal growth factor receptor (EGFR) and give rise to EGFR-positive transit-amplifying C cells that in turn proliferate to generate CD24-expressing migrating neuroblasts. Fluorescence-activated cell sorting (FACS) using a combination of markers such as CD24, GFAP, and EGFR has been used to purify activated stem cells, transit-amplifying C cells, and neuroblasts (Pastrana et al., 2009). Cells that express both EGFR and GFAP are activated B1 stem cells that can be eliminated by an antimitotic treatment (Pastrana et al., 2009). In contrast, GFAP-positive cells that do not express EGFR may be either quiescent stem cells or SVZ niche astrocytes. These cell populations can be further distinguished by the expression of transmembrane glycoprotein, CD133 (prominin), which is present on the primary cilia of neural progenitors (Mirzadeh et al., 2008; Beckervordersandforth et al., 2010; Codega et al., 2014). Using a combination of these markers, it has been shown that quiescent B1 stem cells do not express nestin, an intermediate filament protein considered to be a marker of NSC (Codega et al., 2014). The quiescent state of B1 cells is actively maintained by the GPCR ligands S1P and PDG_2_ (Codega et al., 2014), whereas BLBP is more restricted to active NSC (Giachino et al., 2014).

Adult NSC display regional and temporal specificity in generating different types of bulbar interneurons (Merkle et al., 2007; Batista-Brito et al., 2008; Fuentealba et al., 2015). Based on viral labeling of NSC in distinct spatial SVZ sub-regions, it has been shown that dorsal NSC generate mostly superficial GC and dopaminergic tyrosine hydroxylase (TH)-positive PG, whereas ventral NSC produce deep GC and calbindin-positive PG (Merkle et al., 2007). Calretinin-positive GC and PG, as well as four new subtypes of bulbar interneurons, are mostly derived from NSC located in the anterior and anterior-ventral tip of SVZ, respectively (Merkle et al., 2007, 2014). A lineage tracing method consisting of introducing a large library of 24-base-pair oligonucleotide barcodes into the cells recently revealed that regional specification becomes evident at mouse embryonic stage E11.5, which is maintained from the embryonic to the adult stage (Fuentealba et al., 2015).

This regional heterogeneity is determined by the combinatory action of morphogens and transcriptional factors. For example, lineage tracing of the Emx1 and Gsh2 transcriptional factors, which are highly expressed in the embryonic cortex ventricular zone and the lateral ganglionic eminence, respectively, has shown that Emx1 progenitor cells reside in the adult dorsal SVZ and generate calretinin-positive superficial GC, whereas NSC derived from the Gsh2 lineage produce calbindin-positive interneurons and very low numbers of calretinin-expressing cells (Young et al., 2007). Deep GCs are derived from the Nkx2.1 domain in the ventral SVZ (Delgado and Lim, 2015), whereas four new types of interneurons derived from the anterior-ventral tip of the SVZ are generated from microdomains patterned by the Nkx6.2 and Zic family of transcriptional factors (Merkle et al., 2014). Transcriptional factor Pax6 has a dual role and is required for generating neuronal progenitors and also for directing them toward dopaminergic PG (Hack et al., 2005). In contrast, zinc finger transcription factor Sp8 is required for the specification of calretinin and gabaergic/nondopaminergic PG (Waclaw et al., 2006). NSC in the adult SVZ also generate a small number glutamatergic juxtaglomerular cells in the OB that is specified by the expression of the Neurog2 and Tbr2 transcriptional factors expressed in the subset of progenitors in the dorsal SVZ (Brill et al., 2009). All these studies have established the repertoire of transcriptional factor codes along the dorso-ventral and rostro-caudal SVZ as well as the regional specification of adult NSC in the generation of interneuronal diversity in the OB. In addition to these transcriptional factor codes, morphogens also play an important role in the regional heterogeneity of adult NSC. A study on reporter mice with labeled sonic hedgehog (Shh)-responsive cells revealed that this morphogen gradient plays a general role in the ventro-dorsal specification of NSC (Ihrie et al., 2011). Shh-responsive cells express GFAP and give rise primarily to interneurons in the deep GC layer of the OB (Ihrie et al., 2011), similar to what has been observed after viral labeling of NSC in the ventral SVZ (Merkle et al., 2007). The removal of Shh reduces the production of ventrally derived OB interneurons. Conversely, the ectopic activation of Shh by the expression of Smo2, a constitutive active receptor in dorsal NSC, generates cells that are normally produced by progenitors in the ventral SVZ (Ihrie et al., 2011). Interestingly, a transient domain of Shh in the dorsal SVZ has also been identified, but this domain produces many cells with an oligodendrocyte lineage (Tong et al., 2015). The dorsal domain of SVZ is also determined by persistent Wnt/β-catenin signaling that specify NSC to an oligodendrocyte lineage (Azim et al., 2014a,b). Indeed, live-imaging and single cell tracking of adult NSC and their progeny has revealed that oligodendrogliogenic and neurogenic NSC constitute two distinct lineages that display different responsiveness to Wnt signaling (Ortega et al., 2013).

In addition to regional heterogeneity, adult NSC also display temporal specificity. Batista-Bristo et al. used inducible genetic fate mapping of Dlx1/2 precursors to analyze the precursors of seven OB interneuron subtypes, from embryogenesis through adulthood (Batista-Brito et al., 2008). They found that the production of calbindin-positive interneurons reaches a maximum during late embryogenesis and subsequently decreases postnatally, whereas the opposite is observed for the production of calretinin-positive cells (Batista-Brito et al., 2008). Parvalbumin-positive interneurons in the external plexiform layer are produced perinatally, while Blanes cells, which are bulbar interneurons that provide feed-forward inhibition in GC (Pressler and Strowbridge, 2006), are produced during embryogenesis (Batista-Brito et al., 2008). They also showed that the production of TH-positive cells in the glomerular layer peaks during early embryogenesis and decreases thereafter. However, it has also been demonstrated that TH-positive PG are mainly produced by postnatal NSC (De Marchis et al., 2007).

The production of different subtypes of interneurons from B1 cells is thus regionally and temporally specified. Regional specification allows embryonic information to be maintained, from neuroepithelial to radial glial then to B1 cells, whereas temporal specification controls the order and subtype of the interneurons generated. The precise interplay between temporal and regional factors remains to be determined, as does how the generation of specific interneuronal subtypes is regulated by the behavioral state of animals.

### Non-neurogenic astrocytes

**Figure 1:**
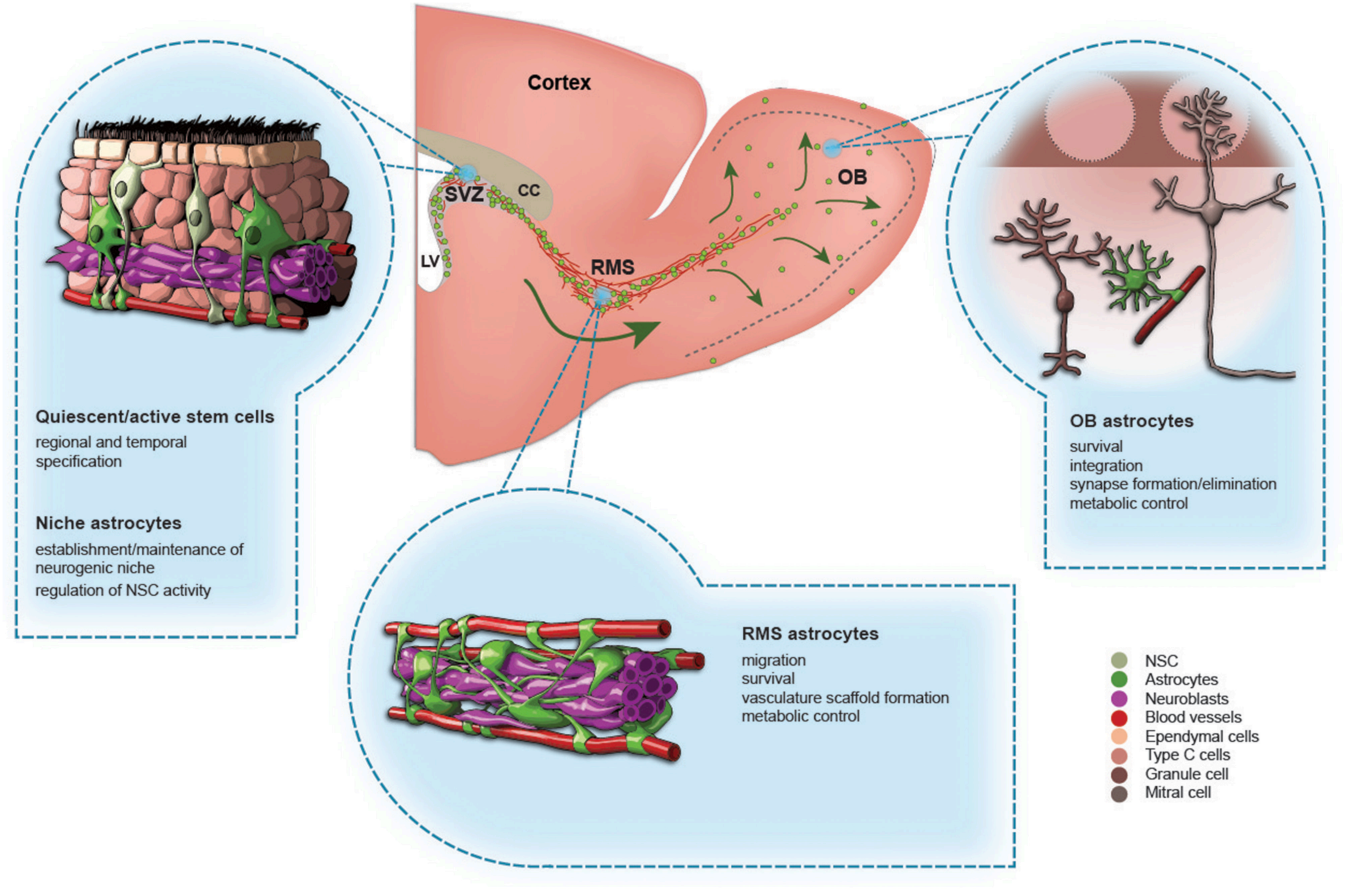
**The role of astrocytes in the adult olfactory bulb neurogenesis**. A schematic drawing of the adult mouse forebrain and illustrations showing different types of astrocytes in the subventricular zone (SVZ), rostral migratory stream (RMS), and olfactory bulb (OB). The functions of each type of astrocytes are indicated.

NSC are found in specialized microenvironments and are exposed to a variety of factors from the cerebral spinal fluid via small apical process bearing a non-motile primary cilium. NSC also contact blood vessels via basal processes (Figure 1; Doetsch et al., 1999; Mirzadeh et al., 2008). Transplanting NSC/progenitor cells into the SVZ of another animal generates OB interneurons, whereas transplanting NSC into non-neurogenic zones limits their neurogenic potential (Alvarez-Buylla and Lim, 2004). These results suggest that the SVZ microenvironment plays an important role in the maintenance of the neurogenic properties of NSC.

In addition to vasculature support and factors present in the cerebrospinal fluid, non-neurogenic astrocytes in the SVZ contribute to the establishment and maintenance of the neurogenic microenvironment. Culturing dissociated perinatal or adult SVZ NSC on astrocyte monolayers has shown that direct contact between astrocytes and SVZ precursors support the proliferation of these precursors (Lim and Alvarez-Buylla, 1999). Similarly, astrocytes from the hippocampus promote the proliferation and neuronal fate of stem cells from the dentate gyrus (Song et al., 2002). This shows that GFAP-positive cells can be sub-divided into two distinct populations, that is, those that are stem cells and those that have non-neurogenic properties but that contribute to the establishment of the SVZ microenvironment.

Labeling strategies based on the use of multiple markers (Codega et al., 2014) or on split-Cre technology (Beckervordersandforth et al., 2010) have been used to discriminate between those two cell populations, as well as other SVZ cells. In the case of split-Cre technology, the coincident activity of GFAP and prominin1 promoters enables the selective labeling and manipulation of adult NSC from non-neurogenic astrocytes (Beckervordersandforth et al., 2010). A transcriptomic analysis of these cells compared to other SVZ cells and non-neurogenic astrocytes revealed enrichment in genes involved in neuronal lineage priming, cilia biogenesis/function, and Ca^2+^-dependent pathways (Beckervordersandforth et al., 2010). Calcium waves have been observed in the SVZ, and it has been suggested that they may define the communication network between NSC and niche astrocytes (Lacar et al., 2011). In line with this, GFAP-positive cells in the SVZ express the gap junction protein connexin 43 and display functional coupling involving 50–60 cells (Lacar et al., 2011).

Morphogens such as BMP, Notch, Wnt, and Shh are also involved in the interplay between non-neurogenic astrocytes and NSC. Shh released by astrocytes stimulates adult neural progenitors to reenter the cell cycle (Jiao and Chen, 2008). GFAP-positive cells in the SVZ express Notch 1 receptors as well as its ligands (Givogri et al., 2006). Notch signaling implies cell-cell interactions and mice deficient in Dlk1, a Notch ligand delta-like homolog, present deficits in postnatal neurogenesis in the SVZ (Ferrón et al., 2011). Dlk1 is secreted by niche astrocytes, whereas its membrane-bound isoform is present in NSC and is required for the inductive effect of secreted Dlk1 on the self-renewal of NSC (Ferrón et al., 2011). BMP signaling is also active in adult NSC (Colak et al., 2008). The conditional deletion of Smad4, a transcriptional factor that mediates BMP signaling, reduces neurogenesis and leads to increased numbers of oligodendrocytes (Colak et al., 2008). BMP ligands and their receptors are expressed by niche astrocytes (Peretto et al., 2004) and ependymal cells express noggin, a BMP feedback inhibitor (Lim et al., 2000). This suggests that there is a complex multicellular interplay between niche astrocytes, ependymal cells, and NSC in the regulation of BMP signaling in the adult SVZ. Wnt canonical, β-catenin-dependent, and non-canonical planar cell polarity (PCP) pathways have been detected in the SVZ (Hirota et al., 2015). While Wnt/β-catenin controls the proliferation of NSC and type C cells, the Wnt/PCP pathway regulates the proliferation, migration, and differentiation of neuroblasts (Hirota et al., 2015). In line with this, Wnt/β-catenin signaling activity has been detected in GFAP-positive astrocytes and Mash1-positive cells using reporter mice and stabilization of β-catenin promotes NSC proliferation (Adachi et al., 2007). Niche astrocytes promote NSC expansion and proliferation via WNT7A (Moreno-Estellés et al., 2012), and the activation of canonical Wnt signaling stimulates the oligodendrogliogenic lineage in SVZ (Ortega et al., 2013). Likewise, EGF signaling affects the proliferation of NSC and type C cells (Doetsch et al., 2002; Pastrana et al., 2009) and promotes oligodendrogenesis at the expense of neurogenesis (Aguirre and Gallo, 2007; Aguirre et al., 2007). Astrocyte-derived factors such as the pro-inflammatory cytokines IL-1β and IL-6 promote NSC neuronal differentiation, whereas insulin-like growth factor binding protein 6 (IGFBP6) and decorin inhibit it (Barkho et al., 2006). It has been also recently demonstrated that quiescent NSC express higher levels of tetraspanin CD9 than parenchymal astrocytes (Llorens-Bobadilla et al., 2015). Thus, while several lines of evidence show that GFAP-positive cells in the SVZ can be sub-divided into NSC and niche cells, the relationship between them remains to be investigated. The use of specific tools to selectively identify and manipulate these two populations will help to pursue this goal. Interestingly, after brain injury, a subpopulation of reactive astrocytes may acquire stem cell properties in response to Shh signaling (Sirko et al., 2013). This shows that the delimitation between neurogenic and non-neurogenic astrocytes is not fixed and may be dynamically modulated. To understand the mechanisms underlying the activation of quiescent NSC, single cell sequencing of two neurogenic regions of the adult mice (Llorens-Bobadilla et al., 2015; Shin et al., 2015) has shown that the downregulation of glycolic metabolism, Notch, and BMP signaling, combined with the concomitant upregulation of lineage-specific transcriptional factors, is required for the entrance of dormant NSC into the primed-quiescent state before they are activated (Llorens-Bobadilla et al., 2015). After an acute ischemic injury, dormant NSC enter into a pre-active state via interferon gamma signaling and are subsequently activated (Llorens-Bobadilla et al., 2015). Interestingly the downregulation of glycolic metabolism (Gascón et al., 2016) and the forced expression of neurogenic transcriptional factors (Berninger et al., 2007; Heinrich et al., 2010) have also been shown to be required for the direct reprogramming of astroglia from cortex into neurons. While it remains to be determined how similar non-neurogenic astrocytes and quiescent NSC are at the level of single cell transcriptomics and what their responses are to different microenvironmental signals, these results suggest that there are similarities in the molecular signatures of these cells.

## Astrocytes in the rostral migratory stream

GFAP-positive astrocytes in the RMS are derived from embryonic radial glia cells (Alves et al., 2002). Some of these cells maintain stem cell properties and can generate different types of OB interneurons (Gritti et al., 2002; Merkle et al., 2007; Alonso et al., 2008). At the structural level, RMS astrocytes have elongated morphology, with their branches aligned along blood vessels and chains of migrating neuroblasts (Figure 1; Peretto et al., 2005; Whitman et al., 2009). Astrocytes undergo extensive reorganization in terms of their structural arrangement and expression of molecular cues postnatally (Pencea and Luskin, 2003; Peretto et al., 2005; Bozoyan et al., 2012). During early postnatal development, astrocytes are located at the borders of the RMS, where they orchestrate the proper development of the RMS (Peretto et al., 2005; Bozoyan et al., 2012). These glial cells synthesize and secrete vascular endothelial growth factor (VEGF), which controls blood vessel formation and growth (Bozoyan et al., 2012). The vasculature scaffold is, in turn, used by neuroblasts to migrate toward the OB (Snapyan et al., 2009; Whitman et al., 2009; Bozoyan et al., 2012). During postnatal development, astrocytes thus regulate the proper development of the migratory stream which takes place at the borders of the RMS. In contrast, the core of the RMS is mostly static and is devoid of proliferative and migrating cells (Pencea and Luskin, 2003; Bozoyan et al., 2012). It remains to be determined how this particular cellular organization with blood vessels, neuroblasts, and astrocytes is transferred into the center of the RMS. We have previously proposed that overall brain growth during postnatal development leads to the elongation and thinning of the RMS. This results in the collapse of the center and the maintenance of the outer border of the RMS until adulthood (Bozoyan et al., 2012).

During adulthood, astrocytes ensheath chains of neuroblasts, and several studies have shown that they play a pivotal role in neuroblast migration (Lois and Alvarez-Buylla, 1994; Snapyan et al., 2009; Kaneko et al., 2010). Neuroblasts cultured on RMS or OB astrocyte monolayers display robust migration, unlike neuroblasts cultured on cortical astrocytes. These differences are regulated by astrocyte-derived non-soluble factors (García-Marqués et al., 2010). In addition, RMS astrocytes release soluble melanoma inhibitory activity (MIA) protein, which is required for neuroblast migration (Mason et al., 2001). The disruption of the glial tube in mutant animals leads to defects in neuroblast chain migration (Chazal et al., 2000; Anton et al., 2004; Belvindrah et al., 2007; Kaneko et al., 2010). For example, neuroblast chain migration is promoted by β1 integrin, and a deficiency in β1 integrin leads to glial tube disorganization and ectopic migration of neuroblasts into the surrounding tissue (Belvindrah et al., 2007). Massive gliosis is observed in neural cell adhesion molecule (NCAM)-deficient mice, which lead to neuroblast migration defects. Astrocytes also express high affinity γ- aminobutyric acid (GABA) transporters whereas neuroblasts express GABA. The inhibition of GABA uptake reduces neuroblast migration (Bolteus and Bordey, 2004). The release of GABA from neuroblasts also induces Ca^2+^ fluctuations in astrocytes, leading to the insertion of high affinity TrkB receptors into the plasma membrane of astrocytes (Snapyan et al., 2009). This, in turn, traps migration promoting vasculature-derived brain derived neurotrophic factor (BDNF), which leads to the entrance of migratory cells into the stationary phase (Snapyan et al., 2009). Astrocytes also release glutamate to control the migration and survival of neuroblasts expressing NMDA receptors (Platel et al., 2010). All these studies suggest that molecular and functional modifications in astrocytes modulate neuroblast migration. Astrocytes may also undergo rapid structural changes to sustain faithful neuroblast migration. Neuroblasts secrete diffusible protein Slit1 whose receptor Robo is present on astrocytes (Kaneko et al., 2010). Astrocytes respond to the repulsive activity of neuroblast-derived Slit, leading to the formation of structurally permissive glial tunnels that enable neuronal migration (Kaneko et al., 2010).

These findings suggest that a complex tripartite interplay occurs between neuroblasts, astrocytes, and endothelial cells in the RMS. When assessing the role of specific molecular pathways in the RMS, it is thus important to consider astrocytes, neuroblasts, and blood vessels as a whole and not as “isolated” units. This will make it possible to refine our understanding of the cellular and molecular pathways that modulate the formation and maintenance of the RMS and neuronal migration. In addition, RMS astrocytes may be a heterogeneous population of cells (Peretto et al., 2005; Whitman et al., 2009; Larriva-Sahd, 2014), and further research is thus required to define the various sub-types of astroglia and their role in the RMS.

## Astrocytes in the OB

Following their arrival in the OB, neuroblasts mature and establish connections in the bulbar network. This occurs initially by receiving axo-dendritic inputs and later through dendrodendritic synapses to the principal neurons (Whitman and Greer, 2007; Panzanelli et al., 2009). The OB has a high density of astrocytes (Bailey and Shipley, 1993), but their role in the maturation and integration of newborn cells remains largely unexplored. Astrocytes may have various functions in the brain network, ranging from the modulation of synaptic transmission and synaptogenesis (Piet et al., 2004; Verkhratsky and Nedergaard, 2014) to structural dynamics, transmitter uptake, and the repair of brain lesions (Correale and Farez, 2015). Interestingly, it has been shown recently that the synaptic integration of newborn hippocampal neurons is locally controlled by astrocytes (Sultan et al., 2015). Blocking vesicular release from astrocytes results in a lower spine density of newborn cells, but only on the neuronal dendrites intersecting the domains of manipulated astrocytes (Sultan et al., 2015). Since the astroglial presynaptic sheath covers the majority of synapses in the adult brain (Verkhratsky and Nedergaard, 2014), it is likely that these cells also play an important role in the formation, maintenance, and/or elimination of synapses in the adult OB.

Astrocytes may also coordinate neuronal metabolism and blood flow by controlling vasodilation and vasoconstriction in response to increased neural activity (Takano et al., 2006). This in turn may affect the survival and integration of newborn cells. Astrocytes control blood flow through the activation of glutamate receptors following intense activity of principal neurons (Takano et al., 2006) or via GABA uptake mechanisms in response to the gabaergic activity of bulbar interneurons (Doengi et al., 2009). This activation triggers an increase in intracellular calcium ions in astrocytes, releasing arachidonic acid and vasoactive metabolites (Otsu et al., 2015). This can happen in the fine processes of astrocytes independently of soma activation, which allows for fine control of blood flow (Otsu et al., 2015) and consequently the local modulation of synaptic units. Astrocytes also provide metabolic substrates from blood vessels to distant neurons through gap junctions (Rouach et al., 2008), which may modulate the morpho-functional properties of newborn neurons farther away.

Astrocytes also control the survival of adult-born neurons via the growth factor-induced release of cytokines (Khodosevich et al., 2013). Connective tissue growth factor (CTGF) is expressed by tufted cells in the OB and enhances the proapoptotic activity of astrocyte-derived transforming growth factor-beta 2 (TGF-β2) (Khodosevich et al., 2013). The release of TGF-β2 from glial cells in turn decreases the survival of PG in an activity-dependent manner (Khodosevich et al., 2013). While the role of astrocytes in the survival, integration, and synaptic maintenance of newborn cells needs to be further studied, these glial cells may modulate the structuro-functional properties of interneurons by both local and long-distance signals. They also are strategically positioned to link activity-dependent changes in blood flow and neuronal metabolism and activity.

In conclusion, astrocytes play a pivotal role in OB neurogenesis. They control the generation, migration, and survival of different subtypes of interneurons as well as the synaptic pruning of newborn cells. Although several key features of astrocytes involved in controlling bulbar neurogenesis have emerged in recent decades, more studies combining new genetic tools, functional imaging, and behavioral tests are required to decipher their role in adult neurogenesis.

## Author contributions

All authors listed, have made substantial, direct and intellectual contribution to the work, and approved it for publication.

### Conflict of interest statement

The authors declare that the research was conducted in the absence of any commercial or financial relationships that could be construed as a potential conflict of interest.
